# Independent associations between health-promoting behaviours, healthcare access, and health-related quality of life in Singapore

**DOI:** 10.1038/s41598-026-49805-4

**Published:** 2026-04-27

**Authors:** Lixia Ge, Yu Juan Lim, Yun Hu, Annie Tan, Yeuk Fan Ng

**Affiliations:** 1 Health Services & Outcomes Research, National Healthcare Group, 1 Mandalay Road, Singapore, Singapore 308205; 2 Saw Swee Hock School of Public Health, National University of Singapore, 12 Science Drive 2, Singapore, Singapore 117549; 3 Khoo Teck Puat Hospital and Yishun Community Hospital, 90 Yishun Central, Singapore, Singapore 768828

**Keywords:** Ageing population, Healthcare access, Health-promoting behaviours, Health-related quality of life, Unmet healthcare needs, Diseases, Health care, Medical research

## Abstract

Population-level evidence on how health-promoting behaviours and healthcare access affect health-related quality of life (HRQoL) in Singapore’s ageing population remains limited. This cross-sectional study examined their independent associations with HRQoL among community-dwelling adults and identified factors associated with complete adherence to health-promoting behaviours and adequate healthcare access. Using 2022 Population Health Survey data from 2,320 participants, adherence to health-promoting behaviours was classified as complete or partial, and healthcare access as adequate or inadequate. Multiple logistic regressions identified associated factors. HRQoL was measured using EuroQol-5 Dimensions-5 Levels (EQ-5D-5L) and EQ Visual Analogue Scale (EQ-VAS). Two-part models for EQ-5D index and generalised linear models for EQ-VAS examined associations with HRQoL. Complete adherence to health-promoting behaviours and adequate healthcare access prevalence was 19.8% and 90.3%, respectively, with divergent associated factors. After adjustment, both variables were independently associated with higher likelihood of perfect health (odds ratio 1.55 and 2.16, respectively) and higher EQ-VAS scores (beta 3.62 and 2.53, respectively), but not with HRQoL among those with imperfect health. Both health-promoting behaviours and healthcare access are independent factors associated with HRQoL. Their largely distinct socio-demographic correlates suggest public health strategies should adopt a dual-track approach: promoting healthy behaviours alongside addressing access barriers to support healthy ageing.

**Supplementary Information:**

The online version contains supplementary material available at 10.1038/s41598-026-49805-4.

## Introduction

Singapore is experiencing a rapid demographic transition and is projected to become a ‘super-aged’ society by 2026, with more than 21% of its population aged 65 years and above^1^. While life expectancy has risen to 83.5 years in 2024^2^, gains in healthy life expectancy have not kept pace, indicating that Singaporeans are living longer but not necessarily healthier^3^. Promoting healthy ageing therefore requires adding years of healthy life and optimising health-related quality of life (HRQoL).

The World Health Organization (WHO)’s framework highlights two important modifiable determinants for healthy ageing: health-promoting behaviours and healthcare access^4^. Health-promoting behaviours such as adequate physical activity, healthy diet, avoidance of tobacco, and adequate sleep help preserve physical and mental capacity while reducing the risk of chronic diseases^5–8^. In parallel, timely and equitable access to quality healthcare services supports early detection and effective management of illness and age-related declines, thereby sustaining well-being and HRQoL. Conversely, barriers to healthcare access, often reflected as unmet healthcare needs, can undermine these benefits^9,10^.

Although often examined separately^5,9^, health-promoting behaviours and healthcare access can function as concurrent determinants along the continuum of prevention: the former supports primary prevention, while the latter underpins secondary and tertiary prevention. Together, they represent interrelated and policy-relevant levers for promoting healthy ageing across the life course.

Guided by the WHO Social Determinants of Health framework, both health-promoting behaviours and healthcare access can be viewed as intermediary determinants linking upstream social factors to downstream health outcomes^11^. Individuals facing both behavioural and structural disadvantages, such as low engagement in health-promoting behaviours and limited healthcare access, may experience compounding risks of poorer HRQoL. Hence, it is valuable to examine health-promoting behaviours and healthcare access jointly as distinct and co-occurring pathways through which modifiable disadvantages may simultaneously influence HRQoL.

Despite growing policy attention through initiatives such as the Healthier SG strategy^12^, population-level evidence on association between healthcare access and HRQoL in Singapore is limited. Inadequate healthcare access often arises from affordability, accessibility, or acceptability barriers^10^ and is consistently linked to poorer health outcomes elsewhere^9,13–16^. Yet, no population-based study has examined the prevalence or impact locally, even amid ongoing healthcare reforms and transition in care delivery models.

Prior international research suggests that age may modify these, as age-related function decline can reduce physical function and increase healthcare needs^17,18^. However, it remains unclear whether these patterns persist in Singapore’s unique context—a high-performing healthcare system with a rapidly ageing, multi-ethnic population.

This study aimed to (1) identify factors influencing health-promoting behaviours and healthcare access, and (2) examine the independent associations of health-promoting behaviours and healthcare access with HRQoL among community-dwelling adults in Singapore.

We hypothesised that:

1. The socio-demographic correlates of the behavioural factor (health-promoting behaviours) and the structural factor (healthcare access) would be largely distinct, consistent with their differing underlying mechanisms of influence; and
2. Both health-promoting behaviours and healthcare access would exhibit independent, positive associations with HRQoL, after mutual adjustment and controlling for socio-demographic and clinical characteristics.

## Methods

### Study design and data source

This cross-sectional study utilised data from the Population Health Survey (PHS) 2022, which was conducted among community-dwelling adults residing in the northern region of Singapore. The survey methodology has been described in detail elsewhere^19^. In brief, the PHS 2022 was a regional population-based initiative aimed at comprehensively understanding the health and healthcare needs, status, and experience of residents of northern region of Singapore. The recruitment and data collection were carried out between July and December 2022.

### Sampling and participants

Participants in PHS 2022 were selected using a two-stage sampling design. In the first stage, a random sample of household addresses was obtained from the Singapore Department of Statistics. In the second stage, one eligible household member was chosen using a Kish grid method^20^. Invitation letters were mailed to selected households about one month before the study visit. Trained surveyors conducted face-to-face interviews in English, Mandarin, or Malay using structured questionnaires.

**Fig. 1:**
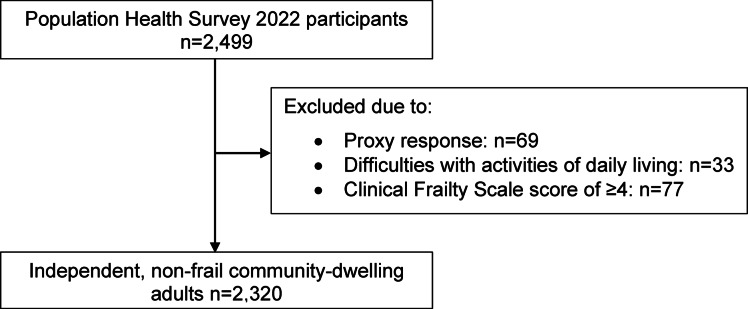
Flowchart on participant selection.

Our analysis was restricted to community-dwelling adults who were independent in activities of daily living and not frail (Clinical Frailty Scale score < 4). Of the 2,499 PHS 2022 participants, 2,320 met these inclusion criteria and constituted the finale analytical sample (Fig. 1).

### Measures

#### Health-promoting behaviours

Modified from prior studies^8,21,22^, health-promoting behaviours in this study were operationalised based on established international and national recommendations and included physical activity, sleep, nutrient intake, smoking, and alcohol consumption.

- Physical activity was defined according to the World Health Organization and Singapore Physical Activity Guidelines as engaging in at least 150 min of moderate-intensity activity, 75 min of vigorous activity, or an equivalent combination per week^23^.
- Optimum sleep duration was defined as 7–9 h per night for adults aged 18–64 years and 7–8 h for those aged 65 years and above, in line with recommendations from the National Sleep Foundation and Health Promotion Board Singapore^24,25^.
- Sufficient nutrient intake was operationalised as consuming two or more sources of protein, two or more servings of fruits or vegetables, and more than five cups of fluids per day.
- Non-smoking was defined as not currently smoking any tobacco products.
- Absence of binge drinking was defined as not engaging in binge drinking (consuming five or more drinks per occasion for men and four or more for women) during the past month, consistent with standard binge drinking thresholds^26^.

A composite health-promoting behaviour score (range: 0–5) was derived by summing the number of domains in which participants met the recommended thresholds. Higher scores indicate stronger adherence. Given the highly skewed distribution of the composite score, with most participants meeting four (41.6%) or all five behaviours (19.8%) and only 1.1% and 7.9% reporting 0–1 and two behaviours, respectively, we dichotomised the score into complete (all 5 behaviours) versus partial adherence (≤ 4 behaviours). This approach allowed us to examine whether meeting all recommended behaviours confers additional health benefits beyond partial adherence, while avoiding sparse cell sizes at the lower end of the distribution. Although dichotomisation may obscure dose-response effects, this operationalisation reflects public health recommendations that emphasise achieving all recommended behaviours for optimal health outcomes.

#### Healthcare access

Adequate healthcare access was evaluated by asking participants whether they had, in the past 12 months, needed but not received a consultation, check-up, or prescribed treatment at any (a) polyclinics/general practitioners or (b) public hospitals. A response of “yes” was classified as having inadequate healthcare access, indicating unmet healthcare needs, whereas “no” or “not applicable (no healthcare need)” responses were grouped as having adequate access as they experienced no unmet need. This self-reported measure has been widely used in prior international studies of healthcare access and equity^18,27^.

#### Health-related quality of life (HRQoL)

HRQoL was assessed using the EuroQol-5 Dimensions-5 Levels (EQ-5D-5L) instrument, which includes five dimensions—mobility, self-care, usual activities, pain/discomfort, and anxiety/depression—each rated on five levels of severity (i.e., no problems, slight problems, moderate problems, severe problems, and extreme problems)^28^. The EQ-5D-5L index scores, ranging from less than 0 (states worse than death) to 1 (perfect health), were calculated using Singapore’s value set^29^. In addition, participants self-rated their overall health using the EQ Visual Analogue Scale (EQ-VAS) ranging from 0 (“worst imaginable health”) to 100 (“best imaginable health”).

#### Socio-demographic and health covariates

Socio-demographic variables comprised age, sex, ethnicity, marital status, highest education level, employment status, and housing type. Age was categorised into young (aged 18–39 years), middle-aged (40–59 years) and older adults (≥ 60 years), reflecting key life-course stages^30^ and aligning with WHO’s definition of older persons^31^ to facilitate cross-country comparisons. In the Singapore context, age 60 is also a relevant threshold for some Singapore health and social initiatives (e.g., Community Health Assist Scheme for seniors, senior outreach by the Silver Generation Office, and community-based health programmes). This categorisation also accommodates the relatively younger demographic profile of the northern region.

Ethnicity was originally categorised as Chinese, Malay, Indian, and Others. Due to the small sample size in the ‘Others’ category (n = 65, 2.8%), it was combined with the ‘Indian’ category for multivariate analyses to ensure adequate statistical power and model stability. Marital status was categorised as single, married, or divorced/separated/widowed. Education level was grouped into: (i) no formal education/primary, (ii) secondary or equivalent, (iii) A-level/diploma, and (iv) degree and above. Employment status was classified as employed, homemaker/housewife, or economically inactive (including retirees, unemployed, and students). Housing type, a reliable socio-economic indicator in Singapore^32,33^, was categorised as: (i) HDB 1/2/3-room flats, (ii) HDB 4-room flats, (iii) HDB 5-room and executive flats, and (iv) private/landed properties.

Health covariates include Body Mass Index (BMI) categories and presence of any chronic disease. BMI was calculated from self-reported height and weight and categorised according to the WHO-recommended Asian-specific cutoffs^33^: (i) normal weight (< 23 kg/m²), (ii) overweight (23 to < 27 kg/m²), and (iii) obese (≥ 27 kg/m²). Chronic disease prevalence was ascertained through self-report of physician-diagnosed chronic conditions, including hypertension, diabetes, hyperlipidaemia, dementia, and other chronic illnesses. These variables were included as covariates given their established associations with HRQoL^30,34,35^.

### Statistical analysis

Descriptive statistics for categorical variables are presented as frequencies and percentages. We compared the distribution of characteristics between groups defined by health-promoting behaviours and healthcare access, respectively, using Chi-square tests. To identify factors associated with each health-related factor, we fitted two separate binary logistic regression models to identify factors associated with (1) health-promoting behaviours and (2) healthcare access. Results are presented as odds ratios (ORs) with 95% confidence intervals (CIs). We then identified common factors across both models to elucidate shared characteristics.

To explore potential effects of coexistence of disadvantages, we examined the overlap and correlation between inadequate health-promoting behaviours and unmet healthcare needs. We cross-tabulated the two variables, computed the proportion of participants experiencing both disadvantages, and estimated the tetrachoric correlation between them.

Between-group differences in HRQoL, presented as mean ± standard deviations (SD), were analysed using Mann–Whitney U tests for EQ-5D-5L index scores and independent samples t-tests for EQ-VAS scores. For multivariable analyses, the EQ-5D-5L index score was analysed using a two-part model^36^ to account for its skewness and substantial ceiling effect. The first part used logistic regression to estimate the probability of reporting perfect health (defined as an EQ-5D-5L index score of 1). The second part used a generalised linear model (GLM) with a gaussian family and an identity link function to model the scores among participants with less-than-perfect health (EQ-5D-5L index score < 1). The EQ-VAS was analysed using a GLM with a gaussian family and an identity link, with results presented as OR or coefficient with 95% CI.

Examination of associations of health-promoting behaviours and healthcare access was guided by theoretical propositions from the integrated framework. Consistent with the WHO Social Determinants of Health framework^11^, we first examined individual associations of health-promoting behaviours and healthcare access with HRQoL (Model 1), followed by joint associations (Model 2) to assess whether they were independently associated with outcomes. All models were conducted in the full study population to estimate overall associations at the population level, adjusted for key socio-demographic covariates (age group, sex, ethnicity, marital status, education, employment, and housing type) and presence of any chronic conditions. Model fit was assessed with likelihood ratio tests and multicollinearity using variance inflation factors (VIFs ≥ 5)^37^. All analyses were performed using Stata SE 17.0 software and statistical significance was set at a two-tailed *p* < 0.05.

#### Sensitivity analysis

To examine whether the association between health-promoting behaviours and HRQoL followed a dose-response pattern, we conducted a sensitivity analysis. In this analysis, the composite health-promoting behaviour score was specified as a three-category variable (0–3, 4, or 5 behaviours) instead of the primary dichotomous variable. All regression models (for both EQ-5D-5L index and EQ-VAS scores) were re-run using this alternative specification.

## Results

### Characteristics of participants

The socio-demographic profile of the 2,320 participants is summarised in Table 1. Briefly, the largest age group was 40–59 years (39.1%), and females (51.8%) slightly outnumbered males. Most participants were Chinese (69.5%), married (69.1%), employed (68.1%), and free of pre-existing chronic conditions (65.5%).

**Table 1:** Characteristics of participants by health-promoting behaviour and healthcare access groups, n (%).

Characteristics	Overall (*N* = 2,320)	Adherence to Health-promoting Behaviours			Healthcare Access		
		Partial (*n* = 1,861)	Complete (*n* = 459)	*p*-value	Inadequate (*n* = 226)	Adequate (*n* = 2,094)	*p*-value
Age group				0.017*			0.001*
18–39 years	747 (32.2)	580 (31.2)	167 (36.4)		89 (39.4)	658 (31.4)	
40–59 years	907 (39.1)	724 (38.9)	183 (39.9)		96 (42.5)	811 (38.7)	
≥ 60 years	666 (28.7)	557 (29.9)	109 (23.8)		41 (18.1)	625 (29.9)	
Ethnicity				< 0.001*			0.535
Chinese	1612 (69.5)	1274 (68.5)	338 (73.6)		164 (72.6)	1448 (69.2)	
Malay	401 (17.3)	353 (19.0)	48 (10.5)		34 (15.0)	367 (17.5)	
Indian	242 (10.4)	186 (10.0)	56 (12.2)		20 (8.9)	222 (10.6)	
Others	65 (2.8)	48 (2.6)	17 (3.7)		8 (3.5)	57 (2.7)	
Sex				0.099			0.566
Male	1118 (48.2)	881 (47.3)	237 (51.6)		113 (50)	1005 (48)	
Female	1202 (51.8)	980 (52.7)	222 (48.4)		113 (50)	1089 (52)	
Marital Status				< 0.001*			0.419
Single	465 (20.0)	346 (18.6)	119 (25.9)		52 (23)	413 (19.7)	
Married	1603 (69.1)	1294 (69.5)	309 (67.3)		153 (67.7)	1450 (69.3)	
Divorced/Separated/Widowed	252 (10.9)	221 (11.9)	31 (6.8)		21 (9.3)	231 (11)	
Employment Status				0.006*			0.384
Employed	1580 (68.1)	1276 (68.6)	304 (66.2)		147 (65)	1433 (68.4)	
Homemaker/housewife	331 (14.3)	278 (14.9)	53 (11.6)		39 (17.3)	292 (13.9)	
Economically inactive	409 (17.6)	307 (16.5)	102 (22.2)		40 (17.7)	369 (17.6)	
Highest Education Level				< 0.001*			< 0.001*
No Formal Education/Primary	379 (16.3)	335 (18.0)	44 (9.6)		22 (9.7)	357 (17.1)	
Secondary or equivalent	716 (30.9)	603 (32.4)	113 (24.6)		57 (25.2)	659 (31.5)	
A-level/diploma	614 (26.5)	471 (25.3)	143 (31.2)		85 (37.6)	529 (25.3)	
Degree and above	611 (26.3)	452 (24.3)	159 (34.6)		62 (27.4)	549 (26.2)	
Housing Type				< 0.001*			0.008*
HDB 1/2/3-room flats	484 (20.9)	430 (23.1)	54 (11.8)		35 (15.5)	449 (21.4)	
HDB 4-room flats	898 (38.7)	745 (40.0)	153 (33.3)		81 (35.8)	817 (39.0)	
HDB 5-room and executive flats	567 (24.4)	438 (23.5)	129 (28.1)		75 (33.2)	492 (23.5)	
Private/landed properties	371 (16.0)	248 (13.3)	123 (26.8)		35 (15.5)	336 (16.1)	
BMI category				0.010*			0.807
< 23 kg/m^2^	976 (42.1)	766 (41.2)	210 (45.8)		94 (41.6)	882 (42.1)	
23 to < 27 kg/m^2^	768 (33.1)	608 (32.7)	160 (34.9)		72 (31.9)	696 (33.2)	
≥ 27 kg/m^2^	576 (24.8)	487 (26.2)	89 (19.4)		60 (26.6)	516 (24.6)	
Presence of any chronic condition				< 0.001*			0.875
No	1520 (65.5)	1183 (63.6)	337 (73.4)		147 (65.0)	1373 (65.6)	
Yes	800 (34.5)	678 (36.4)	122 (26.6)		79 (35.0)	721 (34.4)	

Distributions were compared using Chi-square tests, except for ethnicity, for which Fisher’s Exact test was employed. *p<0.05.

### Factors associated with adherence to health-promoting behaviours and healthcare access

Overall, 19.8% of participants (*n* = 459) reported complete adherence to all health-promoting behaviours. Complete adherence was more common among younger adults (18–39 years: 22.4%; 40–59 years: 20.2% vs. ≥ 60 years: 16.4%), Chinese participants (21.0% vs. Malay 12.0%), single individuals (25.6% vs. married 19.3%), those with higher education (degree and above: 26.0%; A-level/diploma: 23.3% vs. no formal/primary: 11.6%), residents of larger housing (private/landed: 33.2%; HDB 5-room/executive: 22.8% vs. HDB 1/2/3-room: 11.2%), those with BMI < 27 kg/m² (21.5% and 20.8% vs. BMI ≥ 27: 15.5%), and those without chronic conditions (22.2% vs. with chronic conditions: 15.3%) (Table 1).

Multiple regression analysis identified several independent correlates (Table 2). Participants of Malay ethnicity (OR = 0.67, 95% CI: 0.47, 0.95) and those with any chronic condition (OR = 0.70, 95% CI: 0.54, 0.90) were less likely to report complete adherence. Conversely, economically inactive (OR = 1.51, 95% CI: 1.10, 2.07), higher education levels, and residence in larger housing were associated with a higher probability of complete adherence.

**Table 2:** Factors associated with adherence to health-promoting behaviours and healthcare access using logistic regression analyses.

Characteristics	Complete Adherence to Health-promoting Behaviours		Adequate Healthcare Access	
	OR (95% CI)	*p*-value	OR (95% CI)	*p*-value
Age group (ref: 18–39 years)				
40–59 years	1.15 (0.87, 1.52)	0.330	1.21 (0.84, 1.74)	0.295
≥ 60 years	1.22 (0.83, 1.80)	0.310	2.26 (1.33, 3.86)	0.003*
Ethnicity (ref: Chinese)				
Malay	0.67 (0.47, 0.95)	0.026*	1.41 (0.93, 2.16)	0.109
Indians and others	1.25 (0.92, 1.69)	0.153	1.28 (0.83, 1.97)	0.267
Sex (ref: male)				
Female	0.84 (0.67, 1.06)	0.135	1.21 (0.89, 1.64)	0.229
Marital status (ref: single)				
Married	0.77 (0.57, 1.05)	0.094	0.95 (0.63, 1.44)	0.814
Divorced/separated/widowed	0.66 (0.40, 1.09)	0.101	0.75 (0.40, 1.41)	0.371
Employment status (ref: employed)				
Homemaker/housewife	1.13 (0.79, 1.60)	0.511	0.56 (0.37, 0.85)	0.006*
Economically inactive	1.51 (1.10, 2.07)	0.011*	0.76 (0.48, 1.18)	0.221
Highest Education Level (ref: No formal education/primary)				
Secondary	1.41 (0.95, 2.09)	0.090	0.81 (0.47, 1.39)	0.452
A-level/diploma	1.92 (1.24, 2.97)	0.004*	0.49 (0.27, 0.87)	0.016*
Degree and above	2.00 (1.26, 3.15)	0.003*	0.71 (0.38, 1.31)	0.272
Housing Type (ref: public 1/2/3-room)				
Public 4-room flats	1.45 (1.03, 2.05)	0.033*	0.83 (0.54, 1.28)	0.397
Public 5-room and executive flats	2.00 (1.39, 2.87)	< 0.001*	0.55 (0.35, 0.86)	0.009*
Private/landed property	2.83 (1.92, 4.17)	< 0.001*	0.89 (0.52, 1.52)	0.666
BMI category (ref: <23 kg/m ^2^ )				
23 to < 27 kg/m^2^	1.02 (0.80, 1.30)	0.887	1.01 (0.72, 1.42)	0.934
≥ 27 kg/m^2^	0.82 (0.61, 1.11)	0.199	0.88 (0.61, 1.27)	0.498
Presence of any chronic condition (ref: no)				
Yes	0.70 (0.54, 0.90)	0.006*	0.77 (0.56, 1.07)	0.114

*BMI* body mass index, *CI* confidence interval, *OR* odds ratio, *ref* reference. *p-value < 0.05.

Most participants (90.3%, *n* = 2,094) reported adequate healthcare access in the past 12 months. Multiple regression analysis identified several independent factors associated with adequate access (Table 2). Adults aged ≥ 60 years had higher odds of adequate access (OR = 2.26, 95% CI: 1.33, 3.86). In contrast, homemakers/housewives (OR = 0.56, 95% CI: 0.37, 0.85), A-level/diploma holders (OR = 0.49, 95% CI: 0.27, 0.87), and residents of 5-room and executive flats (OR = 0.55, 95% CI: 0.35, 0.86) had relatively lower odds of adequate access (see Table 2 for full results).

The factors associated with each outcome revealed partially distinct patterns. Older age was positively associated with both complete adherence and adequate access. Conversely, higher socio-economic status (indicated by education and housing) was associated with better adherence but was inversely related to adequate access. However, some overlap was observed (e.g., older age was a common correlate), and the overall profiles were not entirely divergent. Nevertheless, the differing directions of association for socio-economic indicators suggest that adherence and access are influenced by partially distinct mechanisms.

### Correlation between health-promoting behaviours and healthcare access

Among participants with inadequate healthcare access, 82.3% reported partial adherence, compared to 80.0% among those with adequate access. This minor difference was reflected in a weak and non-significant tetrachoric correlation (rho = 0.04, *p* = 0.430), indicating that there is little evidence in this sample that these two variables capture overlapping domains of disadvantage, although a non-significant correlation does not prove independence.

### Associations of health-promoting behaviours and healthcare access with HRQoL

Participants with complete adherence to health-promoting behaviours reported a modestly higher mean EQ-5D-5L index score (0.98 ± 0.05) than those with partial adherence (0.97 ± 0.08, *p* < 0.001). Similarly, adequate healthcare access was associated with a higher mean index score (absolute difference: 0.03) compared to inadequate access (0.98 ± 0.07 vs. 0.95 ± 0.11, *p* < 0.001).

Participants with complete adherence to health-promoting behaviours (OR = 1.56, 95% CI: 1.18, 2.08) and those with adequate healthcare access (OR = 2.19, 95% CI: 1.59, 3.01) had higher odds of reporting perfect health (EQ-5D-5L index score = 1, see Model 1 Part 1 in Table 3).

**Table 3:** Associations of health-promoting behaviours and healthcare access with EQ-5D index score.

Exposure	Model 1		Model 2	
	Odds ratio/beta coefficient (95% CI)	*p*-value	Odds ratio/beta coefficient (95% CI)	*p*-value
Part 1: logistic regression (likelihood of perfect health)				
Complete adherence (ref: partial adherence)	1.56 (1.18, 2.08) ^a^	0.001	1.55 (1.16, 2.06) ^c^	0.003
Adequate healthcare access (ref: inadequate)	2.19 (1.59, 3.01) ^a^	< 0.001	2.16 (1.57, 2.96) ^c^	< 0.001
Part 2: GLM for less than perfect health				
Complete adherence (ref: partial adherence)	0.02 (0.003, 0.04) ^b^	0.092	0.02 (0.003, 0.04) ^d^	0.093
Adequate healthcare access (ref: inadequate)	0.02 (0.01, 0.06) ^b^	0.193	0.02 (0.01, 0.06) ^d^	0.188
Combined model				
Complete adherence (ref: partial adherence)	0.01 (0.005, 0.02) ^b^	< 0.001	0.01 (0.005, 0.02) ^d^	< 0.001
Adequate healthcare access (ref: inadequate)	0.02 (0.01, 0.04) ^b^	0.002	0.02 (0.01, 0.04) ^d^	0.002

^a^ Odds ratios for health-promoting behaviours and healthcare access when included in Model 1 separately; ^b^ Beta coefficients for health-promoting behaviours and healthcare access when included in Model 1 separately; ^c^ Odds ratios for health-promoting behaviours and healthcare access when included in Model 2 simultaneously; ^d^ Beta coefficients for health-promoting behaviours and healthcare access when included in Model 2 simultaneously. All models were adjusted for age group, ethnicity, sex, marital status, employment, education, housing type, BMI category, and presence of any chronic condition.

When both exposures were included simultaneously in Model 2, these associations remained significant and stable, with minimal attenuation in effect estimates (Part 1). Among those who reported imperfect health (EQ-5D-5L index score < 1), no significant associations were observed (Part 2). The combined models showed that both complete adherence and adequate healthcare access were positively associated with higher EQ-5D-5L index scores, consistent in both Model 1 and Model 2.

Participants with complete adherence to health-promoting behaviours reported a higher mean EQ-VAS score than those with partial adherence (81.72 ± 9.95 vs. 77.59 ± 11.73, *p* < 0.001). Similarly, the mean EQ-VAS score was higher among participants with adequate healthcare access compared to those with inadequate access (78.68 ± 11.23 vs. 75.81 ± 13.60, *p* < 0.001).

Table 4 summarises the associations of health-promoting behaviours and healthcare access with EQ-VAS scores. In Model 1, when each exposure was examined separately, participants with complete adherence (β = 3.68, 95% CI: 2.62, 4.74) and adequate healthcare access (β = 2.67, 95% CI: 0.91, 4.44) reported higher EQ-VAS scores. When both exposures were included simultaneously in Model 2, the associations persisted with minimal change in the effect sizes (Table 4), indicating minimal confounding between pathways.

**Table 4:** Associations of health-promoting behaviours and healthcare access with EQ-VAS.

Exposure	Model 1^a^		Model 2 ^b^	
	Beta coefficient (95% CI)	*p*-value	Beta coefficient (95% CI)	*p*-value
Complete adherence (ref: partial adherence)	3.68 (2.62, 4.74)	< 0.001	3.62 (2.57, 4.67)	< 0.001
Adequate healthcare access (ref: inadequate)	2.67 (0.91, 4.44)	0.003	2.53 (0.77, 4.29)	0.005

^a^ Health-promoting behaviours and healthcare access were analysed separately; ^b^ health-promoting behaviours and healthcare access were analysed simultaneously in one model. All models were adjusted for age group, ethnicity, sex, marital status, employment, education, housing type, BMI category, and presence of any chronic condition.

Sensitivity analyses using the three-category health-promoting behaviour variable (0–3, 4, or 5 behaviours) confirmed a clear dose–response relationship, with adherence to more health-promoting behaviours associated with increasingly better HRQoL outcomes in all adjusted models (see Supplementary Tables S1 and S2).

## Discussion

### Summary of key findings

Guided by frameworks that conceptualise health as shaped by interrelated behavioural and structural factors, this study demonstrated that both health-promoting behaviours and healthcare access were independently associated with HRQoL among community-dwelling adults in Singapore. Although older age was a common correlate of both domains, health-promoting behaviours and healthcare access were otherwise associated with largely distinct socio-demographic characteristics. This suggests that behavioural and structural advantages or disadvantages do not necessarily cluster within the same individuals in this population.

### Factors associated with health-promoting behaviours and healthcare access

Comparing behavioural (health-promoting behaviours) and structural (healthcare access) factors is informative because they represent largely distinct pathways within the WHO Social Determinants of Health framework, and understanding their different socio-demographic correlates can guide whether integrated or targeted interventions are needed. Our analysis identified partially distinct socio-demographic profiles for adherence to health-promoting behaviours and perceived healthcare access. Nearly 80% of participants did not completely adhere to health-promoting behaviours, underscoring an ongoing public health challenge. Partial adherence was more common among Malay participants, those with lower education attainment, and individuals living in smaller housing units (a proxy for lower socioeconomic status). These patterns may reflect differences in health awareness, financial resources, and opportunities or motivation to engage in healthy behaviours^38–40^.

Notably, individuals with chronic conditions were less likely to report complete adherence to health-promoting behaviours, which is concerning given that this group stands to benefit substantially from sustained lifestyle modification to manage symptoms and improve quality of life^41^. This underscores the need for culturally sensitive, accessible, and condition-specific health promotion strategies that move beyond generic messaging to address the structural and contextual barriers faced by different segments of Singapore’s multi-ethnic population.

In contrast, perceived healthcare access followed a different pattern. Older adults and those with lower education were more likely to report adequate healthcare access, a finding consistent with evidence from other Asian settings^42^ but contrasting with patterns observed in some universal health systems where unmet needs are concentrated among the elderly and socioeconomically disadvantaged groups^10^. Participants aged ≥ 60 years were more than twice as likely to report adequate access than young adults, likely reflecting the protective effect of Singapore’s age-based subsidy schemes (e.g., Pioneer and Merdeka Generation packages), as well as differing expectations of care.

Conversely, higher unmet healthcare needs among younger and middle-aged adults, homemakers, and those with mid-level education may reflect barriers beyond affordability. These include time constraints related to work and caregiving responsibilities, logistical challenges in accessing care during operating hours, and higher services expectations among more educated individuals, who may perceive relatively minor service gaps as unmet needs^15,18^. This group may also be less likely to qualify for substantial government subsidies, increasing sensitivity to cost-related barriers^43^. Together, these patterns suggest that while Singapore’s healthcare system effectively protects older adults from cost-related access barriers, emerging challenges may exist in meeting the evolving needs and expectations of the working-age population. Further qualitative research is warranted to explore these mechanisms, particularly within the evolving *Healthier SG* framework^12^.

### Health-promoting behaviours and healthcare access with HRQoL

Complete adherence to health-promoting behaviours and adequate healthcare access were positively associated with higher HRQoL. The modest absolute differences observed are consistent with the known ceiling effects of EQ-5D measures in general populations^44^ and should be interpreted in terms of population-level impact rather than individual-level change.

Complete adherence to all five health-promoting behaviours was associated with 56% higher odds of reporting perfect health (OR = 1.56) and a nearly 4-point higher EQ-VAS score. Sensitivity analyses using a more granular, three-category behavioural measure further revealed a dose–response pattern, strengthening the conclusion that greater behavioural adherence is incrementally beneficial. This graded association underscores the value of public health initiatives that promote adherence across all recommended behaviours. These findings align with a substantial body of international evidence reinforcing the pivotal role of multi-domain health-promoting behaviours as a cornerstone of healthy ageing^5,41,45^.

This study adds population-based evidence linking inadequate healthcare access to poorer HRQoL in Singapore’s ageing population. Adequate access was associated with a two times higher likelihood of reporting perfect health (OR = 2.19) and a 2.67-point higher EQ-VAS score, underscoring the importance of the structural dimension for HRQoL. This aligns with international studies identifying unmet healthcare needs as a key social determinant of health outcomes^13,15,16^.

The weak and non-significant correlation between health-promoting behaviours and healthcare access indicates that these domains capture largely distinct dimensions of health-related advantage and disadvantage with this population.

### Integrated interpretation and public health implications

By jointly examining health-promoting behaviours and healthcare access, this study integrates behavioural and structural perspectives to inform public health action aimed at improving HRQoL. Health-promoting behaviours primarily represent primary prevention by sustaining intrinsic capacity and functional reserve, while healthcare access facilitates secondary and tertiary prevention through timely diagnosis and effective management of health conditions.

The limited overlap between these domains suggests that improvements in one pathway do not automatically translate into gains in the other. As a result, interventions targeting a single pathway are likely to reach only a subset of the population. Behaviour-focused initiatives (e.g., community exercise programmes and nutritional guidance) and access-oriented interventions (e.g., simplifying subsidy schemes and addressing logistical barriers for working-age adults) are therefore likely to benefit different population segments.

From a systems perspective, these findings underscore the need for integrated public health strategies that simultaneously address behavioural and structural determinants of health. Aligning preventive health promotion with accessible, responsive healthcare services is essential for achieving sustained improvements in HRQoL and supporting healthy ageing at the population level.

### Strengths and limitations

This study has several strengths, including the use of recent population-based data, a theory-driven analytical approach, and robust statistical methods (such as a two-part model) appropriate for the skewed distribution of HRQoL outcomes. It is also among the first studies to jointly examine behavioural and structural factors potentially associated with HRQoL in Singapore’s context.

Several limitations should be acknowledged. First, the cross-sectional observational design limits our ability to establish temporal relationships between exposures and outcomes, constraining causal interpretation. Although key socio-demographic and clinical covariates were adjusted for, residual confounding remains possible. Unmeasured factors such as health literacy, social support, spirituality, personality traits, and time-varying health conditions could potentially influence both health behaviours and HRQoL^46–49^. Reverse causation cannot be ruled out, as individuals with better baseline health may be more capable of engaging in health-promoting behaviours.

Second, reliance on self-reported data introduces potential recall and social desirability bias. Additionally, our alcohol measure captured absence of binge drinking rather than adherence to daily consumption limits; while both are health-relevant, future studies incorporating more detailed alcohol consumption patterns would provide additional insights. Subjective HRQoL measures such as EQ-VAS ratings, may also be susceptible to reporting heterogeneity across individuals due to cultural norms, expectations, language preferences, and reference group effects^50^.

Third, the binary classification of health-promoting behaviours and healthcare access may oversimplify these multidimensional constructs. This limits insight into the distinct associations between specific behaviours or types of unmet needs (e.g., financial, logistical, cultural) and HRQoL. Additionally, our measure of healthcare access grouped individuals with no unmet need and those with no perceived need, potentially misclassifying healthier individuals as having “adequate access”. While this definition follows established practice in health services research, we acknowledge that including individuals without healthcare needs may overestimate the prevalence of adequate access and potentially attenuate the observed association between access and HRQoL. Future studies should adopt more granular measures that distinguish between “no need”, “met need”, and “unmet need” to allow for more refined analyses.

Fourth, the sample was drawn from northern Singapore and restricted to non-frail, community-dwelling adults. While the region broadly reflects national population composition, it includes a higher proportion of younger and working-age residents and a larger Malay population than some other regions. These factors, along with exclusion of frail or institutionalised older adults, limit generalisability to Singapore’s broader ageing population.

Finally, our analyses focused on population-level associations, consistent with the study objectives. Although health-promoting behaviours and adequate healthcare access varied across some socio-demographic subgroups, the small number of participants reporting inadequate healthcare access, as well as limited subgroup sizes, precluded formal assessment of effect heterogeneity.

Our findings should be interpreted considering the post-pandemic context in which data were collected (2022), when pandemic-related lifestyle patterns and healthcare disruptions may still have influenced health behaviours, healthcare access, and HRQoL. Additionally, this study provides a pre-reform baseline before the nationwide implementation of *Healthier SG* in 2023. Future research should re-examine these associations under the new framework to evaluate whether strengthened primary care and preventive support improve health behaviours, access, and quality of life, especially among at-risk groups.

## Conclusions

Both health-promoting behaviours and healthcare access are independently associated with HRQoL among community-dwelling adults in Singapore. Their largely distinct socio-demographic profiles indicate that these factors influence different population subgroups. Public health strategies should therefore adopt a dual-track approach: promoting healthy lifestyles while systematically addressing access barriers, particularly for working-age adults and underserved groups. Future studies conducted under Singapore’s *Healthier SG* reform will be essential to evaluate how integrated primary care models can strengthen both pathways to support healthy ageing.

## Supplementary Information

Below is the link to the electronic supplementary material.

Supplementary Material 1

## Data Availability

According to the Data Protection Act Commission Singapore -Advisory Guidelines for the Healthcare Sector, the personal health data collected for the PHS 2022 are not publicly available due to legal and ethical restrictions related to data privacy protection. However, the minimal dataset underlying the findings in the manuscript are available upon request to interested researchers after authorization of the institutional ethical committee. Interested researchers may contact A/Prof Ng Yeuk Fan (ng.yeuk.fan@nhghealth.com.sg) for data requests.
