# Supplementary material for: Independent associations between health-promoting behaviours, healthcare access, and health-related quality of life in Singapore
Source: Sci Rep. 2026 Apr 27;16:19454. doi: 10.1038/s41598-026-49805-4 (PMC13287698; doi:10.1038/s41598-026-49805-4)
Supplement: Supplementary file 1 — Supplementary Material 1 [file 41598_2026_49805_MOESM1_ESM.docx]

**Table S1.** Associations of health-promoting behaviours and healthcare access with EQ-5D index score

| **Exposure** | **Model 1** | |  | **Model 2** | |
| --- | --- | --- | --- | --- | --- |
|  | **Odds ratio / beta coefficient (95% CI)** | **p-value** |  | **Odds ratio / beta coefficient (95% CI)** | **p-value** |
| **Part 1: logistic regression (likelihood of perfect health)** | | | | | |
| Number of health-promoting behaviours |  |  |  |  |  |
| 0-3 behaviours | Reference |  |  | Reference |  |
| 4 behaviours | 1.43 (1.14, 1.79) ^a^ | 0.002 |  | 1.41 (1.13, 1.76) ^c^ | 0.003 |
| 5 behaviours | 1.89 (1.39, 2.56) ^a^ | <0.001 |  | 1.85 (1.36, 2.52) ^c^ | <0.001 |
| Health access |  |  |  |  |  |
| Inadequate | Reference |  |  | Reference |  |
| Adequate | 2.19 (1.59, 3.01) ^a^ | < 0.001 |  | 2.14 (1.55, 2.95) ^c^ | <0.001 |
| **Part 2: GLM for less than perfect health** | | | | | |
| Number of health-promoting behaviours |  |  |  |  |  |
| 0-3 behaviours | Reference |  |  | Reference |  |
| 4 behaviours | 0.003 (-0.02, 0.02) ^b^ | 0.799 |  | 0.002 (-0.02, 0.02) ^d^ | 0.850 |
| 5 behaviours | 0.02 (-0.003, 0.04) ^b^ | 0.086 |  | 0.02 (0.003, 0.04) ^d^ | 0.092 |
| Health access |  |  |  |  |  |
| Inadequate | Reference |  |  | Reference |  |
| Adequate | 0.02 (0.01, 0.06) ^b^ | 0.193 |  | 0.02 (0.01, 0.06) ^d^ | 0.188 |
| **Combined two-part model** |  |  |  |  |  |
| Number of health-promoting behaviours |  |  |  |  |  |
| 0-3 behaviours | Reference |  |  | Reference |  |
| 4 behaviours | 0.01 (0.001, 0.01) ^b^ | 0.026 |  | 0.01 (0.003, 0.01) ^d^ | 0.039 |
| 5 behaviours | 0.01 (0.01, 0.02) ^b^ | <0.001 |  | 0.01 (0.01, 0.02) ^d^ | <0.001 |
| Health access |  |  |  |  |  |
| Inadequate | Reference |  |  | Reference |  |
| Adequate | 0.02 (0.01, 0.04) ^b^ | 0.002 |  | 0.02 (0.01, 0.04) ^d^ | 0.003 |

*^a^ Odds ratios for health-promoting behaviours and healthcare access when included in Model 1 separately; ^b^ Beta coefficients for health-promoting behaviours and healthcare access when included in Model 1 separately; ^c^ Odds ratios for health-promoting behaviours and healthcare access when included in Model 2 simultaneously; ^d^ Beta coefficients for health-promoting behaviours and healthcare access when included in Model 2 simultaneously. All models were adjusted for age group, ethnicity, sex, marital status, employment, education, housing type, BMI category, and presence of any chronic condition.*

**Table S2.** Associations of health-promoting behaviours and healthcare access with EQ-VAS

| **Exposure** | **Model 1^a^** | |  | **Model 2 ^b^** | |
| --- | --- | --- | --- | --- | --- |
|  | **beta coefficient (95% CI)** | **p-value** |  | **beta coefficient (95% CI)** | **p-value** |
| Number of health-promoting behaviours |  |  |  |  |  |
| 0-3 behaviours | Reference |  |  | Reference |  |
| 4 behaviours | 2.08 (1.03, 3.12) | <0.001 |  | 2.04 (1.00, 3.09) | <0.001 |
| 5 behaviours | 4.81 (3.57, 6.05) | <0.001 |  | 4.74 (3.50, 5.97) | <0.001 |
| Health access |  |  |  |  |  |
| Inadequate | Reference |  |  | Reference |  |
| Adequate | 2.67 (0.91, 4.44) | 0.003 |  | 2.46 (0.72, 4.21) | 0.006 |

*^a^ Health-promoting behaviours and healthcare access were analysed separately; ^b^ health-promoting behaviours and healthcare access were analysed simultaneously in one model. All models were adjusted for age group, ethnicity, sex, marital status, employment, education, housing type, BMI category, and presence of any chronic condition.*
